# Integrating Tummy Time Into Routine NICU Care: Healthcare Workers’ Perspectives on Long‐Term Implementation in India

**DOI:** 10.1002/pri.70376

**Published:** 2026-11-03

**Authors:** Amruta Chutke, Kripa Dholakia, Tanmay Bagade, Puja Padbidri

**Affiliations:** ^1^ Central Research and Publication Unit Bharti Vidyapeeth (DU) Medical College and Hospital Pune Maharashtra India; ^2^ Institute for Physical Therapy Education Widener University One University Place Chester Pennsylvania USA; ^3^ School of Medicine and Public Health University of Newcastle Callaghan Australia; ^4^ Department of Neonatology Bharati Hospital and Research Center Pune India

**Keywords:** developmental care, implementation science, interdisciplinary care, low‐ and middle‐income countries, neonatal intensive care unit, physiotherapy, qualitative research, tummy time

## Abstract

**Background and Purpose:**

Prone positioning for play (‘Tummy Time’) is an evidence‐based recommendation that supports early sensory‐motor development during infancy. Infants who require neonatal intensive care face a heightened risk of motor developmental delays, underscoring the importance of early developmental care practices. Routine implementation of tummy time remains limited in many settings in India and is not included in national guidelines. Socio‐cultural factors further influence the adaptation of such a cost‐effective intervention in India. In response, a structured tummy time practice was introduced at discharge from the neonatal intensive care unit (NICU) of a tertiary‐care hospital in India in 2016. This study explored multidisciplinary NICU healthcare workers' perspectives on implementing tummy time, including its perceived feasibility, acceptability and long‐term integration into routine practice.

**Methods:**

We conducted in‐depth semi‐structured interviews with 12 NICU staff members representing varied professional roles. Interviews followed a predefined schedule, they were audio‐recorded, transcribed verbatim, translated where needed and analysed thematically using MAXQDA software.

**Results:**

We identified the following five themes: knowledge of practice: indications, benefits and precautions; attitudes towards tummy time; family beliefs, caregiver confidence and cultural influences; translating knowledge into practice (staff learning, family coaching and follow‐up) and solutions and strategies (engagement and awareness). The health workers reported being knowledgeable about tummy time and considered it easy to implement. Key barriers centred on parents' lack of confidence in handling their infants. Participants noted that frequent counselling and practical demonstrations can address these barriers. They also highlighted the need for greater awareness in the community and among medical professionals to support consistent practice.

**Discussion:**

NICU staff viewed tummy time positively, considered it feasible to deliver as part of routine care and felt it had become integrated into routine clinical practice 5 years after its initial introduction. These findings support the integration of structured tummy time education and demonstration into standardized NICU discharge pathways in tertiary‐care hospitals in India, alongside strategies to strengthen caregiver confidence and broader awareness among healthcare providers and the community.

## 1 Introduction

Advances in neonatal therapy and the growing integration of anticipatory guidance during transition to home are reshaping early developmental care, with emerging evidence of benefits for long‐term family outcomes (Miller et al. 2024). International public health agencies increasingly highlight tummy time as an essential component of early childhood developmental care, and many now include specific recommendations and parent‐friendly resources in their practice guidelines (American Academy of Pediatrics 2022). Despite this current emphasis, the use of the prone position in infancy has a complex history. During the 1990s, several studies reported an association between prone sleeping and an elevated risk of sudden infant death syndrome (SIDS), leading to widespread guidance urging caregivers to place infants on their backs for sleep (Dwyer et al. 1991; Fleming et al. 1990; Moon et al. 2022; Syndrome 2000).

Although these recommendations effectively reduced SIDS rates, they also resulted in hesitancy among parents to use the prone position even during supervised awake periods. This shift contributed to a rise in positional plagiocephaly, torticollis, and delays in early sensory‐motor development (Efferson and Efferson 2025; Park et al. 2024). In response, public health campaigns refined their messaging by adopting slogans such as ‘Back to Sleep, Tummy to Play’ to distinguish between safe sleep positioning and the developmental benefits of prone play (Moon et al. 2022). Tummy time is now recommended not only for healthy term infants but also for those transitioning from the neonatal intensive care unit (NICU), as evidence supporting its benefits for preterm infants' development is well established (Sampaio et al. 2024).

The recommendation for tummy time is briefly mentioned in the Indian Academy of Paediatrics' *Guidelines on sleep and physical activity* (Guidelines for Parents, n.d.) and *First 1000 days of Parenting‐ Foundations for Life* (IAP ki baat Community ke sath, n.d.). However, these documents do not explain their developmental importance or provide detailed instructions for safe and effective practice. Similarly, national tools such as the *Mother Child Protection Card* advise caregivers to place infants on their stomachs daily but offer limited guidance on implementation, duration, or strategies to support caregiver confidence (Ministry of Health and Family Welfare Ministry of Women and Child Development National Health Mission 2018).

Most evidence on tummy time comes from high‐income countries, where it is routinely incorporated into infant developmental care (Hewitt et al. 2020). In contrast, in India and several other low‐ and middle‐income countries (LMICs), no national guidelines currently provide clear, practice‐oriented guidance on implementing tummy time, particularly for infants transitioning from the NICU to home. Without detailed national guidance, tummy time uptake varies across institutions remains inconsistently embedded in neonatal care pathways, highlighting the need for contextually relevant implementation strategies.

Lack of trained providers, inconsistent support for providers as they change practices, consensus and support from institutional and ground teams to implement the practice, and systems for longterm change are some of the challenges identified in previous studies (Wang et al. 2023). Parents' perspectives, such as anxiety and lower confidence in placing the infant in tummy time (Silva et al. 2023) and lack of knowledge can impact its adaptation in routine practice (Felzer‐Kim et al. 2020). The transition from the NICU to home can be highly stressful for parents, and equipping them with appropriate knowledge and infant‐handling skills can ease this process. Studies examining tummy time have shown positive developmental outcomes when both healthcare providers and parents receive targeted support in implementing the practice (Rodger and Harvey 2025).

While tummy time is widely recommended and its developmental benefits are well established, much of the existing literature focuses on parental adherence, early barriers, or short‐term educational interventions. Far fewer studies have examined how tummy time becomes embedded in routine care within NICUs, particularly in LMIC settings where systems, staffing, family, and cultural dynamics differ from those in high‐income contexts and influence clinical decisions.

In addition, based on the Consolidated Framework for Implementation Research (CFIR), the role of multidisciplinary NICU healthcare workers as long‐term drivers of knowledge translation and practice reinforcement has received limited attention (Reardon et al. 2025). Understanding how healthcare teams perceive, adapt, and continue tummy time practices may offer important insights into the long‐term integration of low‐cost developmental care interventions beyond initial implementation.

Given the established benefits of tummy time for early developmental outcomes, introducing this practice to families as part of age‐appropriate engagement during daily routines is both feasible and valuable. Incorporating tummy time education during the NICU stay also helps prepare caregivers for the transition to home, where consistent practice becomes essential.

In August 2016, we introduced tummy time in the step‐down NICU unit of our tertiary care hospital to train providers and caregivers and embed the practice into routine neonatal care. As frontline implementers, healthcare workers play a central role in translating knowledge into practice. In this study, we explain how a tertiary NICU in India maintained a tummy time programme for 5 years through interdisciplinary ownership, repeated caregiver coaching, and integration into routine workflows. Therefore, this study aimed to explore multidisciplinary NICU healthcare workers' perspectives on the implementation and long‐term integration of tummy time into routine clinical care within a tertiary‐care NICU in India, with particular attention to the organisational, cultural, and contextual factors influencing caregiver uptake and ongoing practice.

## 2 Methods

### 2.1 Study Design and Setting

The study was conducted in a tertiary‐care NICU with access to neonatal physiotherapy services and an established developmental follow‐up programme for high‐risk infants. We used a qualitative descriptive design and conducted in‐depth interviews (IDIs) with healthcare providers working in the NICU of a tertiary‐care hospital in India. We followed the Consolidated Criteria for Reporting Qualitative Research (COREQ) to guide reporting. We conducted interviews 5 years after tummy time was introduced in the step‐down NICU.

### 2.2 NICU Context

The 60‐bed, Level 3B NICU is part of a tertiary care hospital associated with a private medical college and has annual admissions of around 1500, from gestational age of 26 weeks and up. The step‐down unit has about 24 beds, and families can usually stay in the step‐down unit for 1–3 weeks, depending on the infant's age and feeding/medical needs.

### 2.3 Implementation Context: Tummy Time Training and Capacity Building

Tummy time was introduced in August 2016 in the step‐down unit of a Level III NICU as part of anticipatory guidance for developmental care. A neonatal physiotherapist led the roll‐out. Initial training covered neonatologists, fellows, paediatric residents, nurses, a medical social worker and NICU service coordinators, using lectures, mannequin‐based demonstrations, bedside teaching for parents and in‐service sessions for staff. As the practice became embedded, we added short demonstration videos to show safe handling and the expected progression of head control over the first 3 months. After discharge, we revisited the practice during high‐risk follow‐up visits, where families could demonstrate tummy time, receive feedback, and raise concerns. As staff confidence grew, staff delivered training as needed and during the onboarding of new team members. By the time of data collection, tummy time had been part of routine anticipatory guidance in the step‐down unit for several years, allowing participants to reflect not only on initial adoption but also on how the practice was maintained, adapted, and reinforced over time within routine clinical workflows.

### 2.4 Participants and Sampling

Purposive sampling was used to capture perspectives across professional roles. Eligible participants were NICU providers with ≥ 5 months' experience in the unit that were available on the interview day and consented to participate. We excluded floating nursing staff because they cover intermittently and may not have consistent exposure to tummy time. A trained research assistant conducted recruitment and approached participants in person in the NICU/staff room. The interviewer had no supervisory relationship with the participants. All participants provided written informed consent (consent form available in the Supporting Information S1). The final sample comprised 12 participants with role‐based representation aligned to the staffing profile: faculty (2 interviewed), medical residents (2), neonatology fellows (2), nurses (4), social worker (1), and NICU coordinator (1) (Table 1 below).

**TABLE 1:** Proportion of participants in the sample from each profession.

Profession	Total number of staff in the unit	Selected a number of participants for the study
Faculty	4	2
Medical residents	6	2
Neonatology fellows	5	2
Nurses	10	4
Social worker	1	1
NICU coordinator	2	1

### 2.5 Data Collection

The primary investigator (P.P.) developed the interview guide based on a previously validated survey used in psychiatry and reviewed by an experienced clinician‐researcher for clarity and coverage (Andrade et al. 2020). The interviews were semi‐structured and included open‐ended questions about knowledge, attitudes, and practices related to tummy time in the NICU. Interviews were conducted in the NICU staff room by a final‐year MD student trained in community medicine and qualitative methods. The interviewer had no prior relationship with the participants. All participants were anonymised with numerical codes. Interviews lasted about 20 minutes (range: 9–25 minutes) and were conducted in English or Hindi, per participant preference. Basic demographics (qualification, role, NICU experience) were recorded.

### 2.6 Transcription, Translation, and Data Management

We transcribed audio files and translated Hindi interviews into English. The first author checked transcripts for fidelity and translation accuracy. The research team stored de‐identified data and the working codebook on secure institutional servers, accessible only to the team.

### 2.7 Data Analysis

Two qualitative researchers (A.C. and K.D.) conducted the thematic analysis. Transcripts were coded in MAXQDA (v22.8.0); Microsoft Excel was used to organise participant characteristics and to apply descriptive frequency labels. We iteratively developed codes and candidate themes and resolved divergences through discussion. To help readers gauge the relative prominence of perspectives, we used broad frequency bands based on the proportion of participants expressing a view: very few (< 10%), few (10%–30%), some (30%–50%), many (50%–80%), and mostly/almost all (> 80%). These bands are offered as descriptive aids, not inferential statistics.

### 2.8 Reflexivity and Bias Mitigation

To minimise the influence of the PI's prior involvement in establishing the tummy time programme, the PI did not participate in participant recruitment or interviews. Two researchers not involved in programme implementation independently conducted initial coding. The interviewer and researchers (A.C. and K.D.) were independent of clinical supervision. The team discussed assumptions about tummy time (that it is standard developmental care) before analysis and revisited them during coder meetings. The researchers were familiar with the practice but played no role in programme delivery. The team discussed coding decisions, theme development, and interpretation iteratively, and resolved discrepancies through consensus. The CFIR informed the interpretation of findings rather than serving as a coding framework. The researchers paid particular attention to identifying divergent views and examples of ongoing challenges rather than focusing solely on positive experiences. The team maintained an audit trail documenting coding decisions and theme refinement throughout the analytic process.

### 2.9 Thematic Saturation

Data collection and analysis proceeded concurrently. After coding 10 interviews, we identified no substantial new codes. Two additional interviews were conducted across professional groups to confirm thematic completeness and saturation. These interviews reinforced existing categories and provided additional examples rather than generating novel concepts. Therefore, the research team concluded that sufficient thematic saturation had been achieved for the aims of the study.

### 2.10 Ethical Considerations

The Institutional Ethics Committee of the associated Medical College in India approved the study (Approval: BVDUMC/IEC/15 dated 18/10/21). All participants provided written informed consent for participation, audio‐recording, and publication No incentives were offered. De‐identified data were stored on encrypted institutional servers with restricted access and will be retained in line with institutional policy for 5 years after publication.

## 3 Results

We selected interview participants to ensure representation from all professional disciplines in the NICU. Twelve professionals participated in the interview. Demographic data of the participants are shown in Table 2.

**TABLE 2:** Sample characteristics.

Participant number	Qualification	Designation	Number of years' experience in NICU
1	GNM^a^	Senior staff nurse	15
2	MBBS	Third year resident (Peds)	2
3	MD (Peds)	First year fellow (neonatology)	0.8 (10 months)
4	MD (Peds)	Third year fellow (neonatology)	4
5	MD (Peds)^a^	Associate professor	7
6	MSW	NICU coordinator	0.41 (5 months)
7	MBBS	Third year resident (Peds)	2
8	BSc	Staff nurse	1
9	GMN	Staff nurse	1
10	GMN^a^	Staff nurse	4
11	DM (neonatology)^a^	Assistant professor	16
12	MSW^a^	Medical social worker (NICU)	10

^a^ Members present at initial planning and training stages (August 2016).

The themes and codes, along with corresponding quotes from participants, are mentioned in Supporting Information S1: Table S1. Five themes were identified as follows.

### 3.1 Theme 1: Knowledge of Practice: Indications, Benefits, and Precautions

Mostly/almost all participants accurately described how to set up tummy time: an awake and supervised infant on a clear and firm surface, gently rolled into the prone position with the head turned to keep the airway clear. As one nurse put it, *The bedside should be free… and there has to be an attendant present all the time when the baby is on the tummy.* (Participant 1). However, there was some variation in views on when to start. Several felt prone to play could begin soon after birth if medically stable, while others preferred waiting a few days. A senior clinician explained, *Till the time the plastic cord clamp is there, they can put the baby onto the mother's tummy, and after that they have to put it on the floor.* (Participant 11).

Participants commonly described multiple brief sessions per day (e.g., pre‐feed or any tolerated awake period). Perceived benefits included motor activation and early head control, a more symmetrical head shape, bonding, and relief of gas. Reported contraindications included abdominal wall conditions, post‐operative status, invasive ventilation, or medical instability, with caution for very preterm infants or those with hypotonia or failure to thrive.

#### 3.1.1 Role‐Based Contrasts

Nurses most often articulated procedural safety steps (environment set‐up, supervision), whereas physicians and fellows more often referenced developmental milestones and clinical precautions/contraindications. The coordinator and social worker most frequently highlighted family dynamics and caregiver confidence.

### 3.2 Theme 2: Attitudes Towards Tummy Time

Participants described tummy time as simple and beneficial, frequently linking it to earlier head/neck control and broader developmental gains. *Babies who consistently do tummy time reach developmental milestones earlier than those who don't.* (Participant 5). A prevailing view was that barriers can be overcome with patience and repeated counselling: *Some grandmothers do not allow tummy time… But when we explain why we are doing it… they do not refuse anymore.* (Participant 1).

### 3.3 Theme 3: Family Beliefs, Caregiver Confidence, and Cultural Influences

Many participants identified low caregiver confidence, especially maternal worry about breathing, regurgitation, or crying, as the most immediate barrier: *Placing the baby on the tummy needs a little confidence from the mother…* (Participant 7). Some linked hesitancy to cultural norms and advice from elders, favouring supine positioning or caution with very young infants: *Grandparents… with traditional views are reluctant to accept new things.* (Participant 12).

#### 3.3.1 Barrier‐to‐Uptake Pathway

Participants often described a recurring sequence of initial hesitation, limited home practice, follow‐up review, and subsequent re‐counselling and demonstration. Engagement strategies (face‐to‐face positioning, simple toys and talking) were used to reduce infant distress and caregiver anxiety, and support caregivers practice at home, over time. A few families remained reluctant despite counselling, typically where strong family discouragement persisted; in these cases, staff documented concerns and invited repeat demonstrations at subsequent visits.

### 3.4 Theme 4: Translating Knowledge Into Practice (Staff Learning, Family Coaching, Follow‐Up)

#### 3.4.1 Participant Knowledge

Many participants reported little exposure to knowledge on tummy time in formal tertiary education and described learning on the job through bedside demonstrations, brief classes, and videos. Early apprehension gave way to confidence with repetition: *Initially, I used to feel scared; how should the baby be placed prone, but now I am fine; I can do it easily.* (Participant 10). Over time, skills spread through peer coaching during rounds and routine reinforcement in the unit, contributing to a sense that tummy time had become embedded in everyday care.

#### 3.4.2 Coaching Families

Instruction typically combined explanation, demonstration and return demonstration, tailored to family needs. One clinician summarised the approach: *PT takes classes for parents and caregivers… explains the benefits… shows videos… then the PT asks mothers to do it.* (Participant 9). Nurses and physicians reported reinforcing this teaching at the bedside and during discharge planning.

#### 3.4.3 Monitoring After Discharge

At high‐risk follow‐up visits, clinicians observed parent technique and infant head control as informal markers of home practice, re‐counselling when needed: *In the developmental OPD, doctors check if the baby is achieving milestones like lifting the chin and assess whether tummy time is being done.* (Participant 4).

### 3.5 Theme 5: Solutions and Strategies (Engagement and Awareness)

To improve tolerance, participants suggested playful interaction, face‐to‐face positioning, and toys: *We advise mothers to talk to their babies and use toys to keep them engaged…* (Participant 6). To support wider uptake, participants pointed to awareness gaps in the community and among professionals and recommended brief videos, talks, and workshops: *There is so much need for awareness… even among doctors.* (Participant 4).

## 4 Discussion

Participants consistently described tummy time as an established component of routine developmental care, supported by ongoing staff orientation, interdisciplinary reinforcement, caregiver education and developmental follow‐up. These findings provide insight into factors associated with the long‐term integration of a developmental‐care practice in an LMIC context (Shi et al. 2026). Although parental anxiety, concerns about infant safety, and infant distress during tummy time have been widely reported, our findings suggest that barriers in this setting extend beyond the individual caregiver. Participants frequently described negotiations between parents, grandparents, and other relatives regarding infant‐care practices. In the LMIC context, where infant care commonly occurs within multigenerational family structures, implementation decisions may be influenced by a broader family system rather than by parents alone (Kaur et al. 2024; Nyaloko et al. 2025).

The novelty of this study does not lie in identifying barriers such as caregiver anxiety or concerns about infant safety, which have been reported previously. Rather, it lies in documenting how these barriers were addressed and revisited over time within a multidisciplinary NICU system in an LMIC setting. This provides insight into how developmental‐care practices become embedded and normalised within routine clinical care.

Participants described tummy time as becoming embedded within routine clinical workflows through repeated staff training, interdisciplinary reinforcement, caregiver coaching, and developmental follow‐up. The findings can also be interpreted using CFIR (Reardon et al. 2025). Caregiver anxiety and family influences reflect outer‐setting factors that affect implementation beyond the clinical environment, while staff training, interdisciplinary ownership, and routine follow‐up represent inner‐setting and implementation‐process factors that supported continued integration of tummy time into routine care. Participants consistently described staff training as central to successful implementation. Training appeared to do more than improve knowledge; it increased healthcare workers' confidence to actively coach families and respond to questions about safety, handling, and infant tolerance. Physiotherapists often led demonstrations and role modelled safe techniques, while nurses and doctors reinforced the same messages during routine care and follow‐up visits. This overlap in roles seemed important. When multiple team members supported tummy time, it was no longer viewed as a therapy‐specific activity but as a shared aspect of routine neonatal care.

As has been reported elsewhere, parental fear and lack of confidence in handling small or fragile infants were commonly identified barriers to tummy time (Koren et al. 2010; Silva et al. 2023). Participants also highlighted a broader family‐level influence on infant‐care decisions. In many cases, mothers were not the sole decision‐makers regarding newborn care, and grandparents or other senior family members strongly shaped whether they practised tummy time. These findings indicate that, in settings such as India, where family structures often extend beyond the nuclear household, implementation strategies may be more effective when they engage the wider family rather than targeting parents alone. However, participants did not describe these cultural beliefs as rigid. Instead, they described gradually working through concerns through repeated counselling, practical demonstrations, and, when possible, involving other family members in discussions. In this way, cultural beliefs influenced how tummy time was introduced and explained, rather than whether it was attempted at all.

Although parents often understood the benefits of tummy time, many remained hesitant because of concerns about safety, breathing, or infant distress. These concerns were sometimes reinforced by family elders (grandmothers), who discouraged prone positioning based on past practices or beliefs, similar to what the literature reports (Kaur et al. 2024; Nsiah‐Asamoah et al. 2020). Participants described counselling as an ongoing process rather than a single educational interaction. Reassurance, observation of infant cues, and opportunities for parents to practice under supervision were seen as particularly important. This aligns with earlier work showing that caregiver confidence grows through repeated exposure and engagement (Palmer et al. 2019).

Participants also described how parents often judged tummy time based on their infant's response. If the baby appeared comfortable and alert, parents were more likely to continue the practice; if the baby cried or seemed distressed, they reduced or stopped it. In line with this, participants emphasized face‐to‐face interaction, talking to the baby, and staying close during tummy time as simple yet effective ways to improve tolerance and caregiver confidence.

Although participants uniformly recognized the benefits of tummy time, there was noticeable variation in how they described recommended frequency, duration, and contraindications. This variation is not surprising given the lack of consistent guidance across international recommendations. For example, UK guidance encourages short, frequent periods of prone play (Baby Moves‐Start for Life‐NHS, n.d.), whereas American and WHO guidelines suggest higher cumulative daily durations (American Academy of Pediatrics 2022; World Health Organization 2019). Participants' experiences suggest that clearer, locally agreed guidance may help reduce variability in counselling and improve consistency across staff. Participants felt simple visual resources and short demonstration videos were particularly useful for supporting education when specialist staff were unavailable.

Few studies have explored what helps adopt long‐term developmental care practices years after their introduction. Long‐term implementation work in other areas, such as neonatal sepsis management and *Kangaroo Care*, highlights the importance of continued education and reinforcement over time (Bang et al. 2022; Murthy et al. 2023). Similar patterns were evident here. Participants described follow‐up clinic visits as important opportunities to revisit tummy time, observe caregiver technique, and address new concerns that emerged after discharge. This continuity between inpatient care and follow‐up appeared to help maintain caregiver engagement beyond the NICU stay.

The participants viewed teamwork and shared ownership as important for maintaining tummy time within routine care. Previous studies have highlighted that when tummy time is consistently endorsed during routine interactions, the practice gains legitimacy within the unit (Carter and Busby 2023). This collective approach may be particularly important for low‐cost interventions such as tummy time, which rely on repeated reinforcement rather than specialized equipment or infrastructure.

## 5 Strengths and Limitations

This qualitative descriptive study captures perspectives from a multidisciplinary NICU team using purposive sampling, with interviews conducted by an independent, non‐supervisory interviewer, which likely reduced social desirability bias and encouraged candid responses. The researchers collected data several years after the introduction of tummy time, allowing participants to reflect on longitudinal feasibility in routine practice rather than on initial adoption alone.

Several limitations should also be considered. As participants worked in a unit where tummy time was already established, perspectives may reflect a positive framing of the practice; however, variation and uncertainty in responses suggest that accounts were not uniformly idealized. The study was conducted at a single centre and focused on provider experiences, which may limit generalizability and transferability to other settings. Interviews were conducted in two languages, and although care was taken during translation, some language‐related nuance may have been lost. Findings should also be interpreted in the context of a tertiary‐care teaching hospital with access to a neonatal physiotherapist, established developmental follow‐up services, and leadership support for practice change. These organisational characteristics may have facilitated implementation and may not be present in all NICUs in India or similar LMICs. Consequently, transferability should be considered carefully, particularly for smaller centres with fewer rehabilitation resources or limited developmental care infrastructure.

Because only healthcare workers were interviewed, findings concerning caregiver beliefs and behaviours reflect staff perceptions rather than direct parental experiences. Future studies should include caregivers and extended family members to better understand the factors influencing tummy time uptake. Finally, the study did not assess caregiver adherence or infant developmental outcomes, and findings should be interpreted as reflecting implementation experience rather than clinical effectiveness.

Tummy time was implemented within existing systems, without additional funding or major structural changes. Evidence from other settings suggests that structured tummy time guidance can influence early developmental outcomes (Rodger and Harvey 2025). While our team monitored practice through parental discussions and observation during follow‐up visits, we did not objectively measure how much time infants spent in prone positioning at home. Previous studies have shown that parents tend to overestimate tummy time exposure, especially in preterm infants (Inamdar et al. 2024). Future work could build on these findings by linking caregiver‐reported practice with objective measures and developmental outcomes (Palmer et al. 2019).

## 6 Implications for Physiotherapy Practice

Although the study was conducted in India, several findings may be relevant to other settings seeking to implement developmental‐care interventions. The importance of interdisciplinary ownership, repeated caregiver coaching, integration across inpatient and follow‐up services, and low‐resource educational tools is not unique to tummy time and may also support implementing other developmental care practices. The Indian context highlights how evidence‐based practices can be adapted and maintained even where formal national guidance is limited. This approach can also be applied in other low‐resource countries. Several factors supported the continued integration of tummy time within routine NICU care, including leadership support, physiotherapist‐led education, reinforcement by nurses and physicians, integration into discharge counselling, and ongoing developmental follow‐up. Together, these processes helped embed tummy time within everyday clinical practice and may offer practical implementation lessons for other NICUs seeking to integrate developmental‐care interventions.

Physiotherapists can support tummy time implementation through caregiver education, hands‐on training, recognition of infant cues, and reinforcement during follow‐up visits. Clear, unit‐level protocols support consistent implementation within the NICU by guiding physiotherapy input across staff and shifts. Simple strategies such as brief bedside demonstrations and short audio‐visual resources can help standardize caregiver education in busy clinical settings. Given the influence of cultural norms and extended family advice, physiotherapists should use culturally responsive communication, address common concerns such as breathing or crying, and involve family members where appropriate. These findings suggest that greater attention to practical, skills‐based training in developmental care interventions such as tummy time may be worth considering within the undergraduate and postgraduate physiotherapy education.

Beyond tummy time itself, the findings provide practical insights into how low‐cost developmental‐care interventions can be integrated into routine NICU practice through interdisciplinary ownership, caregiver coaching, and continuity across inpatient and follow‐up care.

## Author Contributions

**Amruta Chutke:** Verification of transcription and translation, validation, data analysis and interpretation, manuscript writing – original draft. **Kripa Dholakia:** data analysis and interpretation, manuscript writing – review and editing. **Tanmay Bagade:** data interpretation, manuscript review, and editing. **Puja Padbidri:** conceptualization, methodology, resources, supervision, manuscript writing – original, review and editing. All authors had access to the data.

## Funding

The authors have nothing to report.

## Ethics Statement

This study was approved by the ethics committee of Bharati Vidyapeeth Medical College, Pune (BVDUMC/IEC/15 dated 18th October 2021).

## Consent

All participants signed informed consent for participation.

## Conflicts of Interest

The authors declare no conflicts of interest.

## Supporting information


Supporting Information S1

## Data Availability

Research data are not shared.
