# Supplementary material for: Integrating Tummy Time Into Routine NICU Care: Healthcare Workers’ Perspectives on Long‐Term Implementation in India
Source: Physiother Res Int. 2026 Nov 3;32(1):e70376. doi: 10.1002/pri.70376 (PMC13730251; doi:10.1002/pri.70376)
Supplement: Supplementary file 1 [file PRI-32-e70376-s001.docx]

Supplementary Material

Table S1: Themes identified with supporting excerpts

| Themes | Excerpts |
| --- | --- |
| Knowledge of practice: indications, benefits, and precautions | Participant 11: Till the time the plastic cord clamp is there, they can put the baby onto the mother’s tummy and after that they have to put it on the floor. It should be done strictly on to the floor, with the yoga mat below and no soft clothing, or no beds, bedsheets and everything on to the, below the baby. It should be done on the hard surface. |
|  | Participant 1: First, we wash our hands, then we remove any nesting or loose clothes in and around the baby. The bedside should be free, there should not be anything to the side of the baby, the baby should not be sleeping awake, and there has to be an attendant present all the time when the baby is on the tummy. |
|  | Participant 2: Prone position is not that comfortable position. So the child tries to get out of that position. In a way he moves his limbs and he tries to lift his head. At tummy time, the mother will be sitting near and interacting with the child. The child tries to concentrate. Tummy time helps in forming the bond with mother, interactions and development. |
|  | Participant 4: It promotes the early achievement of developmental milestones like neck holding and crawling. |
|  | Participant 5: Babies with abdominal surgeries should not undergo tummy time. |
| Attitudes toward tummy time | Participant 5: Babies who consistently do tummy time reach developmental milestones earlier than those who don’t. |
|  | Participant 8: Tummy time helps the baby’s brain for development, otherwise later babies may have problems. It helps with hormone development, it helps with bond development. Baby feeds on milk, medicines are also given, it gets mixed in the stomach and gases are released. It’s good for overall development, it helps increase height, weight and helps bonding. |
|  | Participant 10: I have personally seen that babies who get tummy time are more developmentally sound, and they achieve neck control earlier. |
|  | Participant 1: Some grandmothers do not allow tummy time; they say why should such a small baby be put on the tummy? But when we explain why we are doing it, what are the benefits, then they do not refuse anymore. |
| Family beliefs, caregiver confidence, and cultural influences | Participant 7: Placing the baby on the tummy needs a little confidence from the mother, which is not there initially. So every time we teach them, we push them to do it. But still they don’t have that confidence to put the child on tummy. So that is the main reason that parents are a little scared to put the baby on tummies. |
|  | Participant 12: I think in India what happens is that the grandparents, the grandfather, the grandmother have some different ways of doing things. Not all, but some of the grandparents having traditional views are reluctant to accept new things. |
|  | Participant 2: Parents feel insecure when the baby cries during tummy time and immediately take the baby out of that position. Also, Grandmothers, because of certain cultural values, feel that babies are more comfortable in the supine position. |
| Translating knowledge into practice (staff learning, family coaching, follow‑up) | Participant 3: It was hands-on training conducted in the NICU wards. |
|  | Participant 4: Practical demonstrations were conducted with the babies during sessions. |
|  | Participant 10: Initially, I used to feel scared; how should the baby be placed prone, but now I’m fine; I can do it easily. |
|  | Participant 9: PT takes classes for parents and caregivers. She gives the introduction of tummy time and then explains the benefits and then shows practically with a dummy doll, and actual baby. She tells, how often to do it. She also shows videos on the laptop to all the mothers. Then the PT asks mothers to do it. |
|  | Participant 1: I learned quite fast because it’s not very difficult. Madam showed us how to do it one to two times, and then I started doing it. I also teach the mother how to do it. |
|  | Participant 4: In the developmental OPD, doctors check if the baby is achieving milestones like lifting the chin and assess whether tummy time is being done. |
|  | Participant 1: We tell the parents that tummy time should be done at home, too, and when the parents come for follow-up OPD, we check whether they are implementing it or not. So if parents say we are doing it, but we see the baby not moving, then we know that they are not doing it. In such cases, the parents are re-counselled. |
| Solutions and strategies (engagement and awareness) | Participant 6: We advise mothers to talk to their babies and use toys to keep them engaged…..We recommend brightly coloured toys to stimulate the baby during tummy time. |
|  | Participant 4: Mothers vocalize and play sounds to keep babies focused. |
|  | Participant 7: Counselling during follow-ups helps reinforce the practice after discharge. |
|  | Participant 4: I think that awareness should be created about tummy time, like, being a ……., I never knew about it. There is so much need for awareness. These minute things really hold a lot of importance in the developmental care of the baby in later on life. Not all institutes, hospitals focus on this. So I feel that we need to spread a lot of awareness, even among doctors. |
|  | Participant 5: We need more awareness about tummy time, especially in rural areas. |
